# Evaluation of a self-retaining distractor for hip joint arthroscopy in toy breed dogs

**DOI:** 10.1186/s12917-019-1779-y

**Published:** 2019-01-23

**Authors:** Jihye Kim, Jaemin Jeong, Haebeom Lee

**Affiliations:** Department of Veterinary Clinical Sciences, College of Veterinary Medicine, Chungnam National University, Daejeon, 34134 South Korea

**Keywords:** Arthroscopy - distraction - articular cartilage damage (ACD)

## Abstract

**Background:**

Hip arthroscopy has become a viable option over the last few years for small animal orthopedic diseases, including hip dysplasia, osteoarthritis, and joint evaluation. However, the narrow joint spaces make it difficult to manipulate the instrument, and depth of tissues make it difficult to distract the joint space. In addition, it is very difficult to maintain consistent distraction over time with a manual distraction due to hand fatigue. To overcome these difficulties, distractors are used in human medicine to improve safety and accuracy of arthroscopy. Therefore, in this study, distractor devices were applied to hip joints in small toy breed dogs to evaluate their technical efficacy. Potential iatrogenic neurovascular and articular damage were also evaluated by comparing two techniques for performing hip joint arthroscopy: the self-retaining distractor and external manipulation.

**Results:**

The mean ± SD of the joint distraction distance was 8.88 ± 3.54 mm in the self-retaining distraction group and 2.37 ± 0.82 mm in the manual traction group. As the joint space increased, surgeons could more easily place an arthroscopy portal and more comfortably manipulate the instrument with a distractor device. Furthermore, the acetabular cartilage damage (*p* = 0.004) was significantly greater in the external manipulation group, but articular damage to the femoral head (*p* = 0.940) was similar in both groups.

**Conclusion:**

The results of this study revealed that use of a distractor device can be a viable option for performing hip arthroscopy in small animals. The device significantly improved the surgeon’s performance without surgical assistance, and it reduced iatrogenic cartilage damage compared with manual traction. Further study is needed to quantify neurapraxia associated with distractor placement.

## Background

Hip arthroscopy has become a viable option over the last few years for small animal orthopedic diseases, including hip dysplasia, osteoarthritis, joint evaluation, fracture repair, and bone fragment removal. The procedure offers the advantages of decreased postoperative pain, increased visualization, low morbidity rates, and increased precision [1–3].

Hip joint arthroscopy is commonly used in human medicine. It is a complementary diagnostic tool but is superior to computed tomography or magnetic resonance imaging in assessing articular cartilage [4, 5].

However, arthroscopic examinations in small animal hip joints are more difficult because of the relatively smaller joint space makes arthroscopic manipulation difficult. In addition, manipulating instruments in this limited space during arthroscopic procedures can damage the articular cartilage; thus, good visibility should improve safety and efficiency in performing arthroscopic exams. Increased joint space may offer better visualization and instrument manipulation as well as reduce cartilage damage [6, 7].

To secure sufficient joint space, the hip should be tractioned and simultaneously adducted and rotated internally during arthroscopy. Extra assistance may be needed for manual traction to improve visualization, which increases fatigue.

To overcome these issues, surgeons can use several traction devices that eliminate the need for assistants. These are commonly used when the consistent distraction forces to maintain increased joint space are limited by the assistant’s ability. The devices may also provide the surgeon greater freedom of movement [1, 2].

Several studies have demonstrated the efficacy of distractor devices, and most surgeons advocate their use during arthroscopy; these devices are commonly used to secure joints in human medicine [8–10].

Similarly, the use of distractors to perform arthroscopy in animals was studied in several joints to evaluate the technical feasibility and efficacy [3, 7, 11]; however, to the author’s knowledge, most of these reports were associated with medium to large breed dogs, and they did not perform arthroscopic examinations of hip joint with distractors in toy breed dogs.

Although hip joint disorders, such as hip dysplasia and osteoarthritis, are more common in large-breed dogs, they also occur in breeds of other sizes. Any age or breed can be affected [1]. Therefore, arthroscopy may be a valuable diagnostic tool for small animals with hip joint disorders.

This study evaluated the efficacy of using a distractor versus manual traction by assessing visualization, the difficulty of procedure, and the degree of iatrogenic articular damage during hip arthroscopy in toy breed dogs.

We hypothesized that the procedure would be easier for the surgeon and that there would be less iatrogenic articular cartilage damage in the group receiving the joint distractor than in the group receiving external manipulation.

## Results

### Distraction length

Distraction length was measured by lateral radiography. The length was significantly longer in the distractor group than in the external manipulation group. The mean distraction length ± SD was calculated as 2.37 ± 0.82 mm in the external manipulation group and 8.88 ± 3.54 mm in the distractor group (*p* < 0.001).

### Time required for arthroscopic procedures

The arthroscopic procedure time was 198.35 ± 113.73 s in the distractor group and 150.60 ± 78.38 s in the external manipulation group (*p* = 0.329). The additional time required to apply the external distractor was 60.40 ± 15.57 s.

### Visualization and difficulty of procedure score

The visualization scale during hip arthroscopy did not significantly differ between the two groups (*p* = 0.231). All hip joint areas were well-visualized in both groups.

During the procedure, 4/20 cases (20%) experienced instrument interference between the distractor and arthroscope, making it difficult to visualize the caudal portion; therefore, hip joints were slightly flexed and rotated internally for better visualization.

The arthroscopic portal installation score differed significantly between the two groups (*p* = 0.015) and was lower in the external manipulation group than in the distractor group. In the distractor group, 16 cases (80%) received excellent scores, whereas only 8 cases (40%) received excellent scores in the external manipulation group. The number of arthroscopic slippage during the procedure was much higher in the external manipulation group than in the distractor group, and the difference was statistically significant (*p* = 0.035) (Table 1).

**Table 1:** Difficulty of procedure score during hip joint arthroscopy

Group	Arthroscopic slippage score			Portal installation score		
	Poor	Good	Excellent	Poor	Good	Excellent
Distractor (*n* = 20)	1	2	17	0	4	16
	5%	10%	85%	0%	20%	80%
External manipulation (*n* = 20)	2	9	9	3	9	8
	10%	45%	45%	15%	45%	40%

### Articular damage to the acetabulum and femur head

The mean % articular cartilage damage to the acetabulum was 0.00 ± 0.00% with the distractor and 0.01 ± 0.01% using external manipulation. The mean % articular cartilage damage to the femur head was 0.01 ± 0.01% with the distractor and 0.01 ± 0.04% with external manipulation. After India ink staining, the articular cartilage to the acetabulum was significantly damaged in the external manipulation group (*p* = 0.004); however, no significant differences in femur head damage was observed between the two groups (*p* = 0.940).

### Bone fracture

Post-operative radiographs and gross examination also revealed no fracture or fissure line of bones associated with K-wire placement.

### Iatrogenic sciatic nerve damage

The result of disarticulation revealed that there was no gross lesion for sciatic nerve injury by K-wire insertion or distraction.

## Discussion

Joint manipulation performed by an assistant during arthroscopy to overcome the limited joint space in toy breed dogs may induce fatigue as the procedure progresses. This study demonstrated that using a distractor for hip joint arthroscopy in toy breed dogs instead of assisted manipulation may be a viable alternative. Use of the distractor device reduced iatrogenic cartilage damage and significantly improved the surgeon’s ease in performing the surgery. The surgeon was better able to perform procedures in the distractor group due to the increased joint space. Increased joint space length was significantly higher by approximately 4 times in the distractor group. As mentioned previously, the increased joint space increased the ease of manipulating the arthroscope and navigating the intraarticular joint [12, 13]. The number of trials required to install the arthroscopic portal and the chances of failed arthroscopy during the procedure in the distractor group were lower than those in the external manipulation group. As a result, the ACD% of the acetabulum in this study was also lower (*p* = 0.004) in the distractor group than in the external manipulation group. However, the femoral head ACD% was not significantly different between the two groups.

In a previous report, the feasibility of using a distractor device was evaluated based on joint space distraction length using a special device to accurately load a specific force, but these studies did not perform additional arthroscopic examination [3]. Unlike previous studies, our study was performed in a situation similar to that in a clinic, where surgeons applied a distractor device during hip arthroscopy without assistants, and the increased joint space length, arthroscopic visualization and surgeon’s ease in performing the procedure were assessed.

The intraarticular visualization score and total arthroscopy time were not significantly different between the two groups (*p* = 0.329, *p* = 0.231). This result may be attributed to the use of normal hip joints and observation of the basic intraarticular structures in our study. In patients with hip joint disease, thickened joint capsules would make arthroscopic manipulation more difficult; thus, the examination time would likely increase. An additional time of approximately 1 min (60.40 ± 15.57 s) wass required to install the distractor. Despite this extra time, the operating time would not be substantially increased. Use of the distractor can quickly become a standard procedure if the surgeon is proficient, and the assistant’s fatigue may be reduced [14]*.*

Despite the increased joint space, instrument interferences occurred between the distractor and the arthroscope in approximately 20% (*n* = 4) of the distractor group. These interferences made it difficult to visualize the caudal hip joint in most cases. However, the surgeon adjusted by slightly flexing the hip joint and rotating it internally, thus all parts could be well-visualized.

Non-invasive arthroscopic distraction methods are commonly achieved by another surgical team member. The advantage of these methods is that they rapidly alter the joint position; however, the assistant’s inadvertent movement and fatigue, which may affect joint positioning and stability during the procedure, are unavoidable [2, 15]. Invasive techniques using the joint distractors could distract directly the joint to be examined without involvement of another joints, whereas non-invasive techniques, such as manual traction, may apply forces at the distal limb; therefore, not only targeted joint but also another joints will be involved. Furthermore, as a sterilized distractor device can be used, the surgeon can take the device into the operative field independently and easily control it during arthroscopy.

However, complications such as distraction-related pain, bone fracture, nerve and soft tissue injuries may occur [3, 10, 14, 16, 17]. According to a human medicine report, pain scores were increased due to traction. However, intra-articular analgesia significantly decreased the pain scores postoperatively. Inaccurate position or excessive size of the penetrating pins may cause an iatrogenic fracture. In human medicine, even 5–6-mm diameter pins resulted in no complications or difficulty in resuming weight bearing [10]. In our study, the size of K-wire used in the distraction technique did not exceed 25% of the bone diameter, and no fractures occurred.

Transient neurapraxia of the pudendal, sciatic, and peroneal nerves caused by the distraction has been reported to occur in 3.1 to 5.2% on humans, but patients recover within several weeks [8, 18]. In particular, the sciatic nerve is anatomically contiguous around the hip joint; therefore, gross examination was performed to identify if there were signs of iatrogenic injury to the sciatic nerve but none was seen [19]. However, as this was a cadaveric experiment, complications such as sciatic neurapraxia related to distraction were difficult to quantify. Also, “Freeze-thaw” processes can potentially alter characteristics. Further in vivo studies are needed to detect related nerve injury in small animal arthroscopy.

## Conclusion

The results of this study revealed that use of a distractor device may be a viable option for performing hip arthroscopy in toy breed dogs. Use of a distractor device significantly improved the surgeon’s ease in manipulating instruments without surgical assistance and reduced iatrogenic cartilage damage compared to use of manual traction techniques; however, neurapraxia or distraction related pain are possible. Further study is needed to quantify the nerve injury associated with distraction.

## Methods

### Specimens and groups

Forty hip joints (*n* = 20 dogs) were obtained from toy breed dogs who were euthanatized for reasons unrelated to this study. None of the dogs had hip joint abnormalities upon radiographic examination. Body weight, body condition score, and breed were recorded. Breeds included mongrel (*n* = 13), poodle (5), Maltese (1), and miniature pinscher (1), with 9 males and 11 females and a mean body weight of 4.3 kg (range, 2.0–7.4 kg).

Cadavers were stored at − 20 °C and thawed at room temperature for 24 h before the procedure. One hip joint side from each of the 20 cadavers was randomly assigned to the distractor or the external manipulation group, and all hip joints were arthroscopically examined.

### Distractor device application

**Fig. 1:**
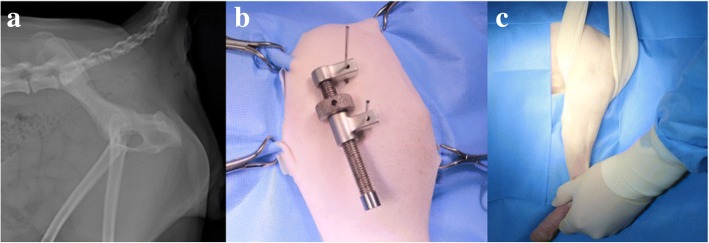
Installation of distractor device. **a** The radiographic image of pelvis. Location of two K-wires placement were marked with yellow star. **b** Distractor device was placed cranially to place arthroscopy portal. **c** External manipulation was performed by an assistant

In the distractor group, two 1.6-mm K-wires were inserted bicortically under the fluoroscopy guide at the lesser trochanter level and the point of intersection 1 cm cranial from the acetabular rim and the midline of the iliac wing width, respectively. The distractor device (External stifle distractor, Veterinary Instrumentation, Sheffield, UK) was then installed (Fig. 1). The distractor device application was stopped when the joint space would not increase further and resistance was encountered. All increased joint spaces were then checked by fluoroscopic examination.

In the external manipulation group, the assistant stood opposite the surgeon and positioned the elastic band (Elastic Bandage-S, Suseong, Korea) over the inguinal region. Grasping it with one hand, the band was simultaneously adducted while the distal hindlimb was internally rotated in the opposite direction.

### Arthroscopic examination

Each cadaver was positioned in lateral recumbency and clipped over the hip joint. To distend the joint space, fluid was infused using a 3-ml syringe perpendicular to the limb at the site just dorsal to the greater trochanter. A 1.9-mm, 30-degree arthroscope was used (Stryker Endoscopy, Stryker, US), and the portal was established using a #11 scalpel blade for the stab incision at the 12-o’clock position. The egress portal was established using 22-gauge intravenous catheter stylets at the 5-o’clock position in the right hip and at the 7-o’clock position in the left hip. Fluid flow was maintained using a pressurized fluid pump (DualWave™, Arthrex, US) with the pressure set at 20 mmHg.

A single surgeon (HBL) performed all arthroscopic procedures. Surgeon’s experiences with arthroscopy included approximately 20 hip arthroscopy procedures in large breed dogs and 100 stifle arthroscopies with distractor in toy breed dogs. Five preliminary practices for hip arthroscopy with distractor in toy breeds were performed on cadavers prior to performing this study. Cranial, caudal, and neutral sections of the hip joint were examined, including the ligamentum teres, femoral head, cranial and caudal joint pouches, acetabulum, acetabular labrum, and the synovial membrane.

### Evaluation

**Fig. 2:**
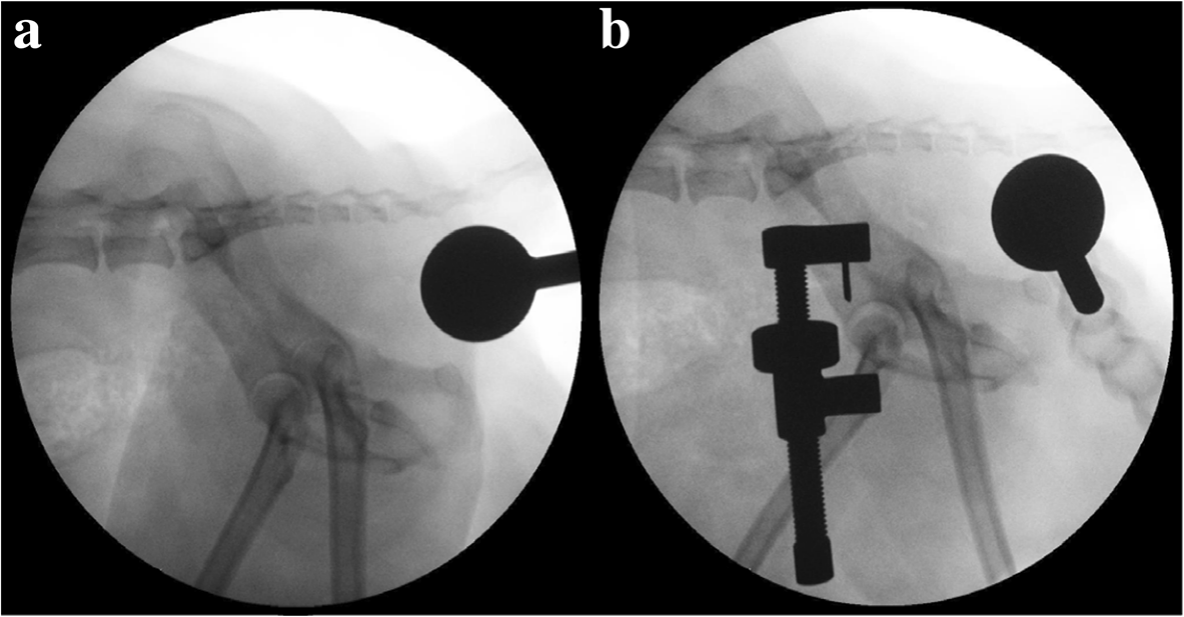
Fluoroscopic image of a hip joint distraction length. **a** External manipulation group, **b** Distractor device group. Measurement was performed to calculate distance between acetabular center and femoral center)

The joint distraction distance of each group was calculated by mediolateral fluoroscopy imaging. All images were obtained by slightly tilting the pelvis until the greater ischiatic notch to be distracted was superimposed with the opposite iliac bone. The hip joint extension angle was determined between the pelvic and femoral axis and was, between 90 and 100°. The magnetic ball with a diameter of 25-mm was positioned at the level of the greater trochanter of the distracted limb (Fig. 2).

Before arthroscopy, the distractor device application time was measured, and the arthroscopy examination time was recorded.

Visualization was scored based on views of each of the cranial, neutral, and caudal portions of the hip joint.

In the neutral portion, three intraarticular structures were observed: the ligamentum teres, the acetabular fossa, and the articular cartilage of the deep and medial femoral head. In both the cranial and caudal portions, the synovial membrane, femoral head cartilage and joint capsule were observed. The visualization scores were graded as follows. If all the structures were seen, the score was ‘excellent’. If 1–3 areas could not be visualized, the score was ‘good’, and when 4–6 or more than 6 areas could not be observed, scores of ‘poor’ and ‘fail’ were given, respectively.

The difficulty of procedure score was evaluated according the following 2 categories; the number of arthroscopic portal installation trials and slippage of the arthroscope from the joint space during the procedure. The score was graded as follows. If the portal was installed on the first or second trial, ‘excellent’ and ‘good’ scores were given, respectively. If installation failed twice or more, it was scored as ‘poor’. Similar to the portal installation scoring system, the number of arthroscopic slippages was graded as follows: no slippage, excellent; one slippage, good; and more than two slippages, poor.

**Fig. 3:**
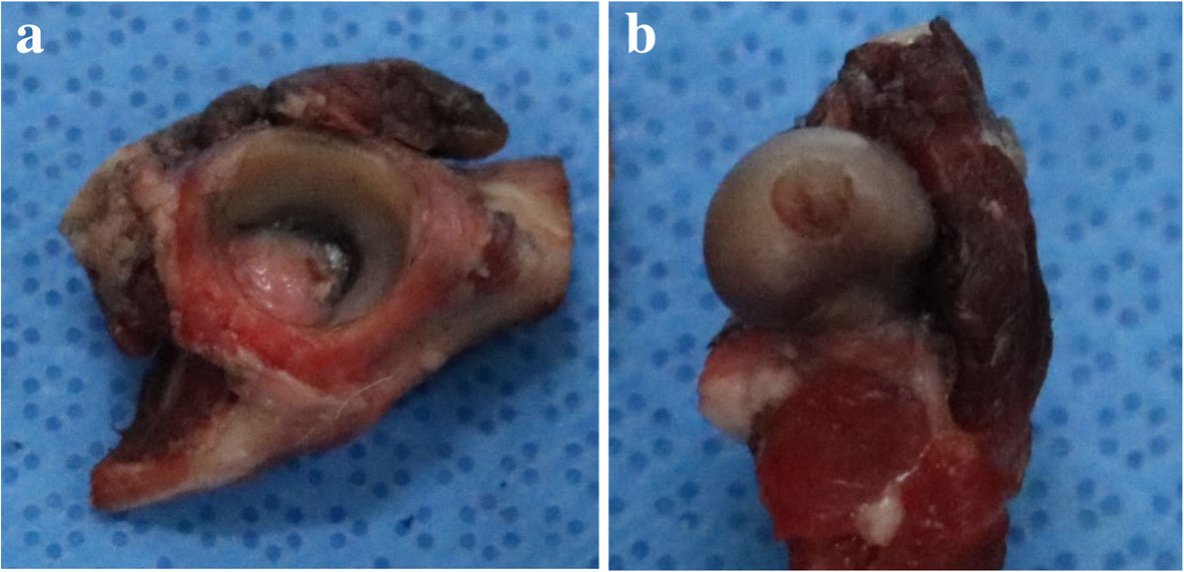
ACD (%) was calculated using computer software program. The percentage area of entire surface of the articular cartilage and damaged articular cartilage for both acetabulum (**a**) and femoral head (**b**)

After arthroscopic examination, the bone fracture or fissure line, and the sciatic nerve injury was also grossly evaluated. All hip joints were disarticulated to remove soft tissue, leaving only the femoral head and acetabular articular surfaces. The femoral head and acetabulum were separated by disarticulating the ligamentum teres. All articular surfaces of the femur head and acetabular cartilage were exposed. Before painting the india ink, the cartilage surface was rinsed in saline. India ink staining of the cartilage surfaces of the femoral head and acetabulum were performed by painting an India ink over the joint surfaces, allowing it to sit for 60 s and then washing in tap water to remove excess ink [20, 21]. The joints were then sequentially photographed to document the weight bearing surfaces of femoral head and acetabulum. All photographs were taken perpendicular to the surface while minimizing light contamination. All digital images were evaluated by ImageJ software (ImageJ ver. 1.50, National Institutes of Health, US) to calculate the degree of the area of cartilage damage (ACD%) in the femoral and acetabular cartilage. For each image, the entire area and stained with India ink were calculated (Fig. 3). ACD% was calculated using computer software. The percentage of the entire articular cartilage surface and damaged articular cartilage for both the acetabulum (A) and femoral head (B)).

### Statistical analysis

All statistical analyses were performed using SPSS software, version 22.0 (IBM SPSS Statistics 22.0, IBM Corp., US). The mean ± SD was calculated for the epidemiologic data, time for distractor placement, and distraction length.

The Mann-Whitney U test was used based on data normality to compare the parameter scores for two different distraction methods (visualization score and difficulty of procedure score) as well as to compare the femoral head ACD% and the acetabular articular cartilage ACD%. *P* < 0.05 was considered statistically significant.

## Abbreviations

ACD: Articular cartilage damage
CT: Computed tomography
MRI: Magnetic resonance imaging
